# Treatment-Resistant Femoral Lymph Node Abscess Caused by Cat Scratch Disease: A Case Confirmed by Polymerase Chain Reaction

**DOI:** 10.7759/cureus.100030

**Published:** 2025-12-24

**Authors:** Seiichiro Inoue, Shinichiro Sumii, Kotaro Araki, Kouki Tomari, Takashi Matsuoka

**Affiliations:** 1 Pediatric Medicine, Okinawa Prefectural Yaeyama Hospital, Ishigaki, JPN; 2 General Pediatrics, Okinawa Prefectural Nanbu Medical Center and Children’s Medical Center, Haebaru, JPN; 3 Division of Clinical Pharmacology, The Hospital for Sick Children, Toronto, CAN

**Keywords:** bartonella henselae, cat scratch disease, femoral lymphadenitis, lymph node, pediatric, polymerase chain reaction

## Abstract

Cat scratch disease (CSD), a zoonosis caused by *Bartonella henselae*, frequently presents as localized lymph node swelling near sites of inoculation, such as scratches or bites. Here, we report a rare pediatric case of CSD in an eight-year-old girl, manifesting as a treatment-resistant femoral lymph node abscess. The patient had a history of chronic exposure to domestic cats. Initial empirical therapy was ineffective, and serology alone was inconclusive. A definitive diagnosis was established by detecting *B. henselae* DNA using polymerase chain reaction (PCR) from lymph node tissue, aspirate, and serum samples obtained during incision and drainage. This case highlights the clinical utility of molecular diagnostic techniques, such as PCR, in atypical CSD presentations and supports their early use to improve diagnostic accuracy, guide appropriate therapy, and avoid unnecessary invasive procedures.

## Introduction

Cat scratch disease (CSD) is a zoonosis caused predominantly by *Bartonella henselae* and is typically transmitted through scratches or bites from domestic cats. Most immunocompetent children develop self-limited regional lymphadenitis within one to three weeks of inoculation, most often in the axillary, cervical, or inguinal regions [1]. Although the clinical course is usually benign, a subset of patients experience suppuration and prolonged fever, which can complicate diagnosis and management [2]. We report the case of an eight-year-old girl with a treatment-resistant femoral lymph node abscess and recurrent lymphadenopathy distant from the presumed inoculation site. A definitive diagnosis was established by polymerase chain reaction (PCR) detection of *B. henselae* DNA from lymph node tissue, aspirate, and serum. This case underscores the diagnostic value of integrating molecular testing with clinical and serologic assessment in atypical pediatric CSD.

## Case presentation

**Figure 1:**
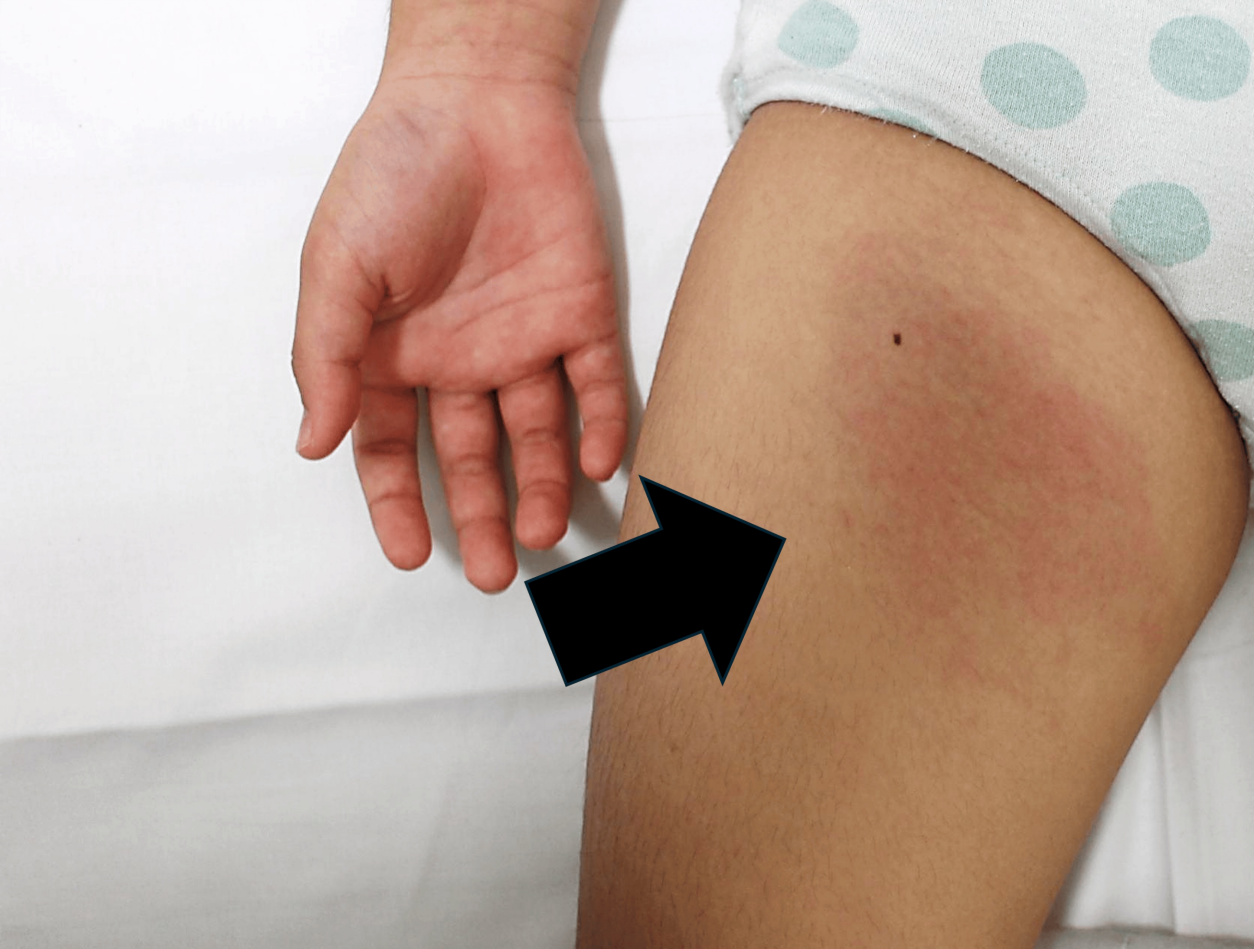
Subcutaneous mass with erythema and tenderness on the anterior aspect of the right thigh. A well-demarcated erythematous area overlying a subcutaneous mass on the anterior right thigh in a pediatric patient was observed; the black arrow indicates the lesion.

An eight-year-old girl with a history of close contact with five domestic cats presented with anterior right thigh pain and a persistent fever of approximately 38°C. She reported frequent cat scratches on her fingers but denied any injuries to her legs. Physical examination revealed a painful, erythematous mass in the anterior right thigh (Figure 1), without lymphadenopathy elsewhere or signs of local trauma.

**Figure 2:**
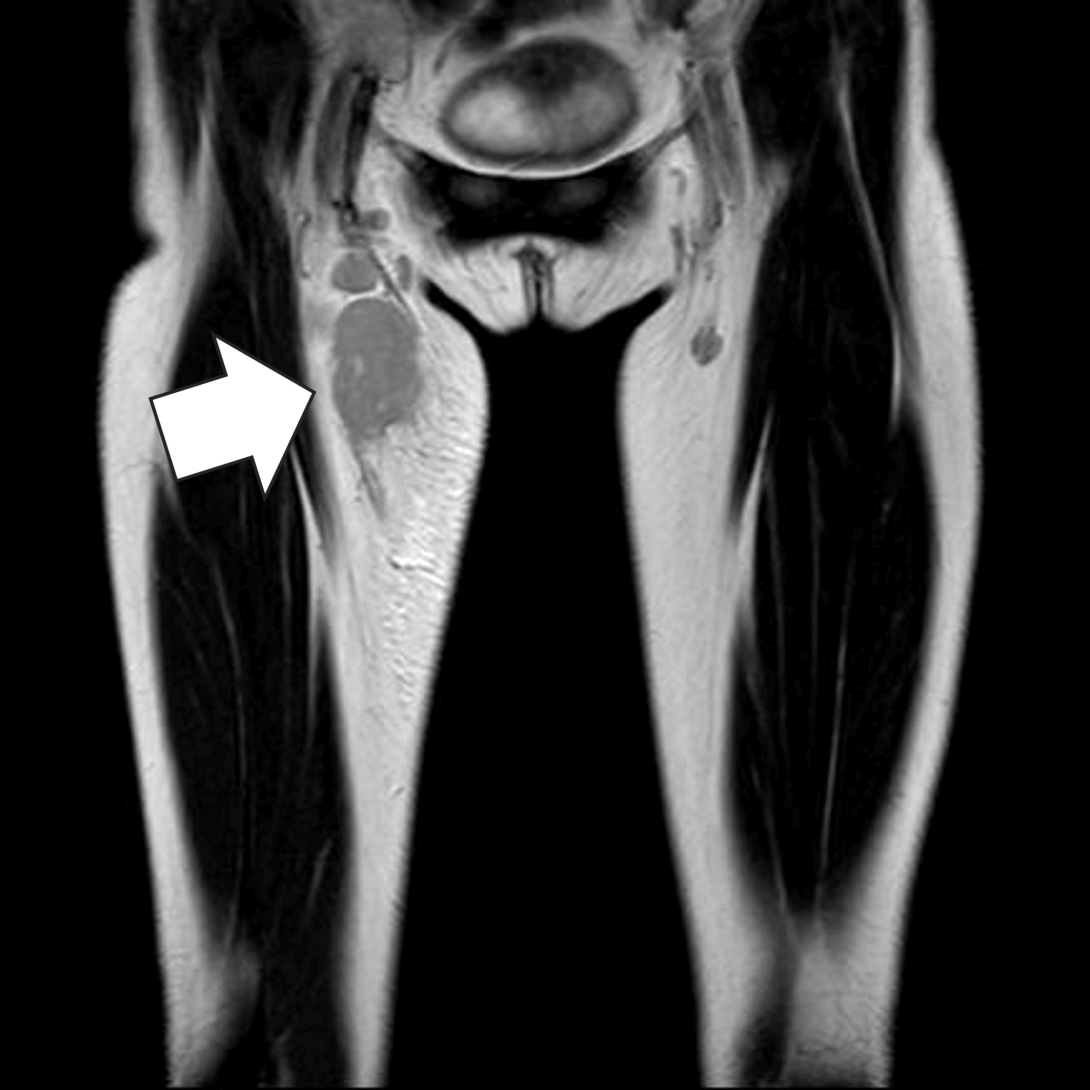
MRI findings of an enlarged lymph node in the anterior right thigh with inguinal lymphadenopathy. Coronal T2-weighted MRI of the pelvis and thighs demonstrating an enlarged, hyperintense lymph node in the subcutaneous tissue of the anterior right thigh, with additional enlargement of inguinal lymph nodes; the white arrow indicates the lesion. Surrounding soft tissue edema is also present.

**Figure 3:**
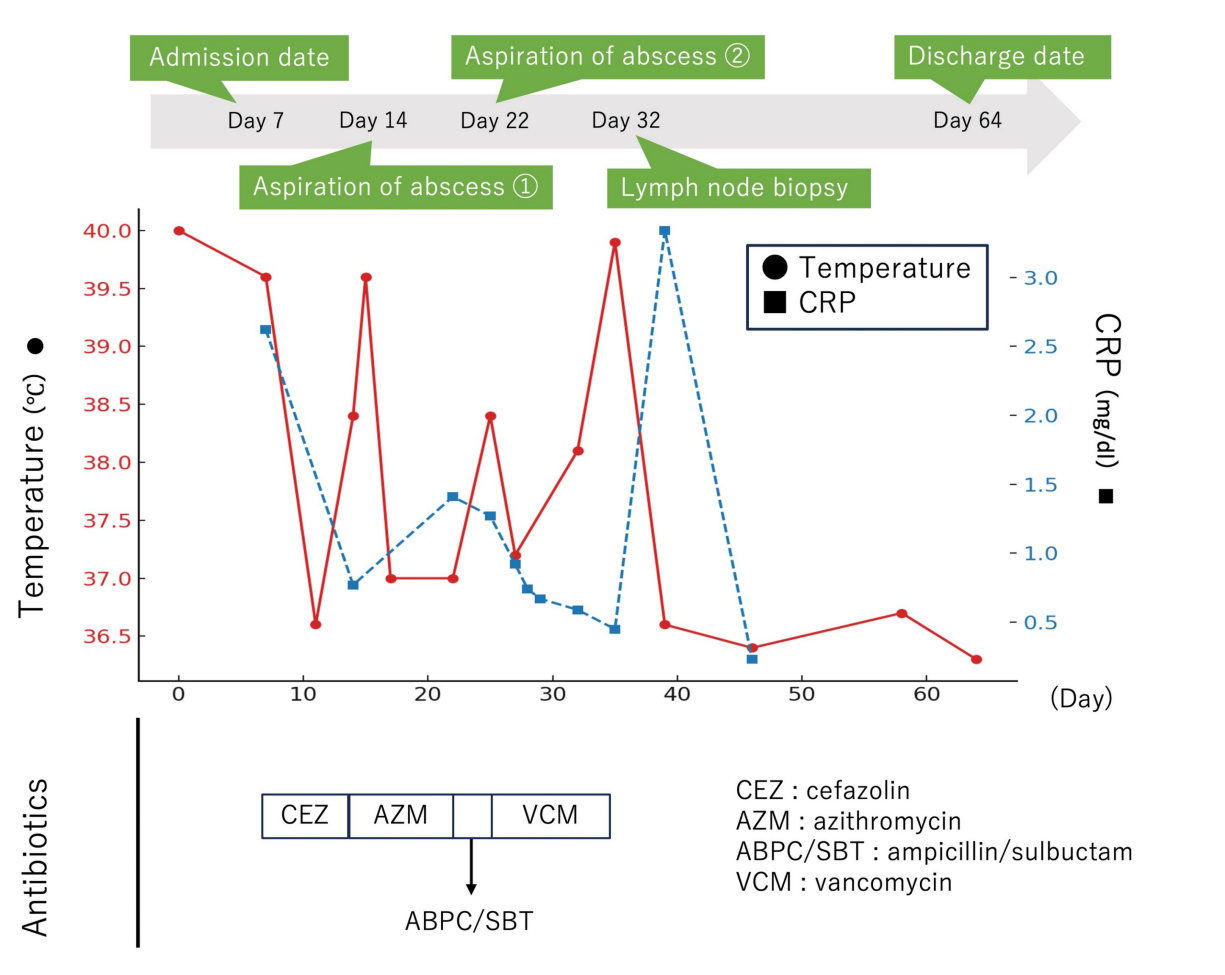
Clinical course showing changes in body temperature and CRP levels during hospitalization. The patient’s body temperature (red circles, left axis) and C-reactive protein (CRP) levels (blue squares, right axis) are plotted over time. Antibiotic regimens are shown at the bottom, transitioning from cefazolin (CEZ) and azithromycin (AZM) to ampicillin/sulbactam (ABPC/SBT), and, subsequently, vancomycin (VCM). Green arrows indicate the key clinical events: hospital admission (day 7), abscess aspiration (days 14 and 22), lymph node biopsy (day 32), and discharge (day 64). A marked improvement in fever and CRP was observed after the lymph node biopsy and abscess aspiration.

Laboratory tests revealed elevated C-reactive protein, erythrocyte sedimentation rate, IgG, and soluble IL-2 receptor levels (Table 1). Repeated blood cultures and cultures of aspirated pus, including those for anaerobic bacteria and fungi, were all negative. Ultrasonography of the thigh showed a 3.5 × 1.9 cm enlarged lymph node without definitive evidence of abscess formation, while MRI demonstrated swelling of femoral and inguinal lymph nodes (Figure 2). Empirical cefazolin was initiated for suspected acute suppurative lymphadenitis and cellulitis, leading to temporary defervescence; however, fever recurred after seven days (Figure 3).

**Table 1:** Laboratory and microbiological findings of the patient. CBC = complete blood count; WBC = white blood cell; RBC = red blood cell; Hb = hemoglobin; Plt = platelet; BUN = blood urea nitrogen; AST = aspartate aminotransferase; ALT = alanine aminotransferase; LDH = lactate dehydrogenase; CRP = C-reactive protein; ESR = erythrocyte sedimentation rate; sIL-2R = soluble IL-2 receptor; GTP = γ-glutamyl transpeptidase; ANA = antinuclear antibody

Parameter	Value	Reference range
CBC with differential		
WBC	9,040 /µL	3,300–8,600/µL
Neutrophils	67.1%	47.0–61.0%
Lymphocytes	23.8%	25.0–45.0%
Monocytes	8.0%	4.0–7.0%
RBC	4.42 ×10^6^/µL	3.86–4.92 × 10^6^/µL
Hb	12.5 g/dL	11.6–14.8 g/dL
Plt	245 × 10^3^/µL	158-348 × 10^3^/µL
Biochemical tests		
BUN	6.0 mg/dL	6–20 mg/dL
Creatinine	0.36 mg/dL	0.29–0.53 mg/dL
Na	136 mmol/L	138–145 mmol/L
K	4.5 mmol/L	3.4–4.7 mmol/L
Cl	102 mmol/L	98–106 mmol/L
AST	25 U/L	16–38 U/L
ALT	12 U/L	4–25 U/L
LDH	361 U/L	286–606 U/L
CRP	2.62 mg/dL	<0.2 mg/dL
ESR	40 mm/hour	5–15 mm/hour
IgA	114 mg/dL	50–301 mg/dL
IgM	178 mg/dL	97–370 mg/dL
C3	205 mg/dL	84–151 mg/dL
C4	24 mg/dL	17–40 mg/dL
CH50	29.4 U/mL	25.0–48.0 U/mL
sIL-2R	614 U/mL	121–613 U/mL
Ferritin	85.7 ng/mL	3.6–114 ng/mL
ANA	<1:40 titer	<1:40 titer
Bartonella serology		
Day 17 after symptom onset		
*B. henselae* IgG	1:512	<1:64
*B. henselae* IgM	1:20	<1:20
Day 39 after symptom onset		
*B. henselae* IgG	1:256	<1:64
*B. henselae *IgM	1:20	<1:20
Other infectious tests		
QuantiFERON-TB	Negative	Negative
Bacterial culture results		
Blood	Negative	Negative
Aspirated pus	Negative	Negative

**Figure 4:**
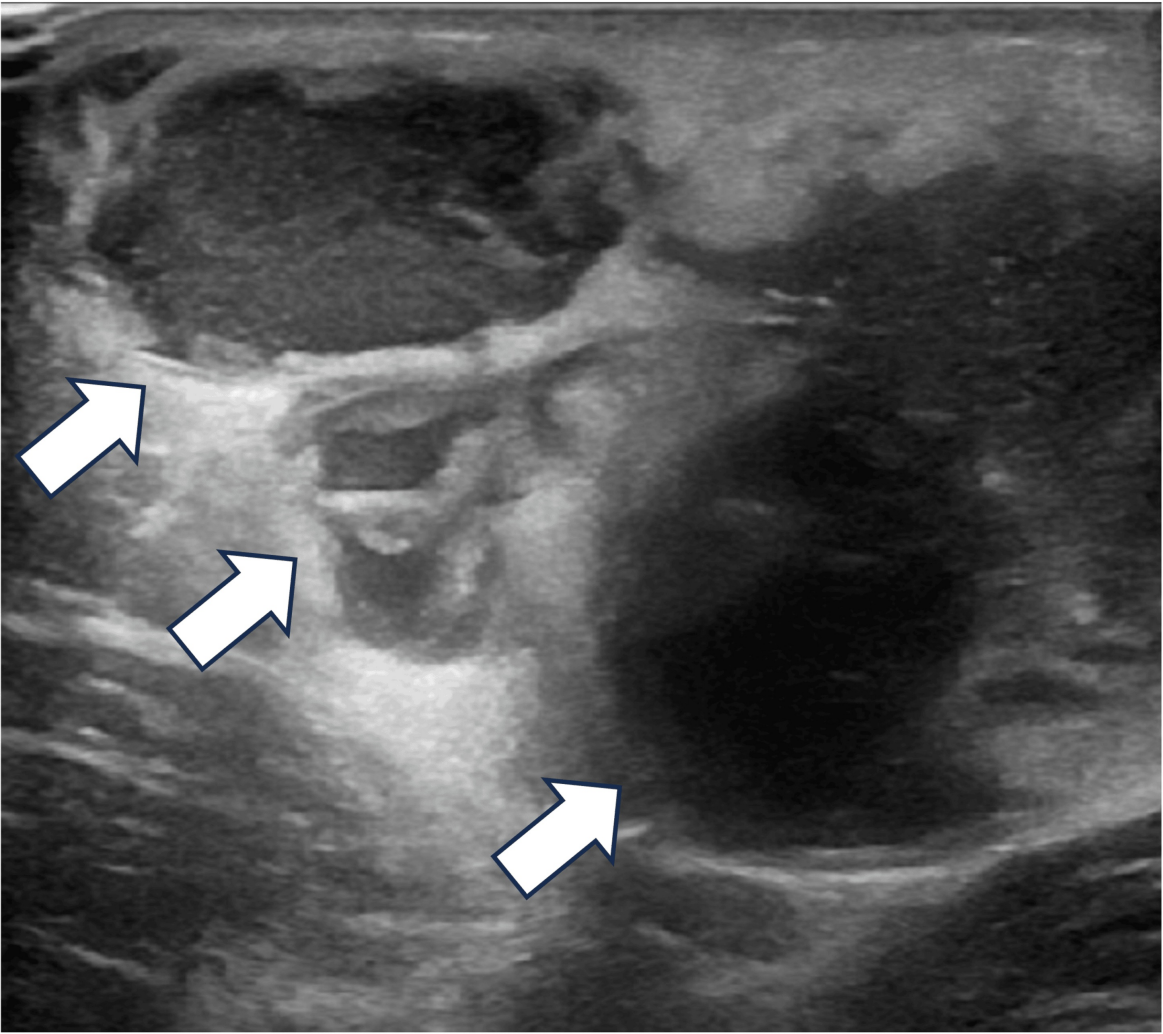
Pre-drainage ultrasonography of the right thigh. Ultrasonography of the anterior right thigh demonstrating a well-defined hypoechoic lesion with internal septations, consistent with an abscess. Surrounding soft-tissue echogenicity suggests inflammatory changes.

By the second week, repeat ultrasonography identified an abscess at the same site, and percutaneous drainage was performed (Figure 4). A more detailed history revealed a recent scratch on her left finger from a domestic cat. Although the lymphadenopathy was distant from the injury site, CSD was suspected, prompting a switch to oral azithromycin, which led to rapid defervescence and marked clinical improvement. She was discharged upon achieving clinical stability.

Seven days after discharge, the patient was readmitted with recurrence of fever and right thigh pain, along with newly developed lymphadenopathy below the right knee. Given the recurrence of symptoms despite azithromycin treatment, multidrug-resistant bacteria or atypical mycobacterial infection were considered. A second drainage procedure was performed, and additional microbiological investigations, including cultures for nontuberculous mycobacteria, were obtained. Antimicrobial therapy was subsequently changed to ampicillin-sulbactam; however, clinical improvement was limited. Because methicillin-resistant *Staphylococcus aureus* infection was then considered, treatment was switched to vancomycin. All repeat cultures, including those for nontuberculous mycobacteria, were negative.

Serological testing in week five revealed elevated *B. henselae* IgG (1:512) and IgM (1:20). However, due to chronic cat exposure, these results could not clearly distinguish between past and active infection. Because symptoms persisted despite multiple drainage procedures, and because exclusion of alternative diagnoses and acquisition of adequate specimens for PCR were required, surgical incision and drainage of the femoral lymph node were performed. Pus, biopsy tissue, and serum samples were submitted for PCR targeting *B. henselae* according to the established protocol [3]. DNA was extracted using the Quick-DNA/RNA Viral Kit for frozen specimens and the Quick-DNA/RNA FFPE MiniPrep Kit for FFPE tissue (both from Zymo Research, USA). Real-time PCR was conducted using a *B. henselae*-specific probe targeting the 16S-23S rRNA intergenic spacer region, in accordance with the methods described by Maggi et al. [3].

All three sample types tested positive for *B. henselae* DNA, confirming that the treatment-resistant abscess and recurrent lymphadenopathy were due to CSD. Although the PCR results did not directly change antimicrobial therapy or fundamentally alter the treatment strategy at that stage, earlier molecular confirmation might have allowed avoidance of prolonged ineffective antibiotic therapy and potentially reduced the need for invasive procedures, such as lymph node biopsy, while still permitting appropriate drainage when clinically indicated. The patient improved rapidly postoperatively and was discharged on day 64, with no recurrence during follow-up.

## Discussion

CSD, caused by *B. henselae*, is commonly transmitted through cat scratches or bites and typically presents one to three weeks after inoculation. It often resolves spontaneously, manifesting as localized lymphadenopathy in the axillary, cervical, or inguinal regions [1]. This case was unusual due to the atypical femoral location of infection and the formation of a treatment-resistant abscess, with repeated recurrences despite antimicrobial therapy. Although CSD is generally considered a benign condition, atypical presentations may pose significant diagnostic and therapeutic challenges, particularly in regions with high cat exposure [4].

Diagnosis of CSD relies on clinical features, exposure history, serological testing, and molecular methods. Indirect immunofluorescence assay (IFA) remains the most commonly used serological tool, with interpretive thresholds generally considered significant at IgG titers ≥1:256 and IgM ≥1:20 [5,6]. However, studies in Japan have shown that seroprevalence varies regionally and seasonally, reflecting the prevalence of cat bacteremia [7]. In regions with frequent cat exposure, baseline IgG titers may be elevated, increasing the risk of overdiagnosis [8]. Okinawa, where this case occurred, is one such region [7].

Seropositivity can reach up to 50% in certain populations, particularly those with frequent contact with cats [7], and asymptomatic infections, including *B. henselae* organisms in red blood cells of asymptomatic blood donors, have been reported [9]. Therefore, serological results must be interpreted with caution, particularly in atypical cases. In this case, although antibody titers met diagnostic thresholds, chronic cat exposure and an atypical clinical course rendered the diagnosis uncertain until confirmation by PCR. When serological findings clearly meet diagnostic criteria in a typical clinical context, PCR testing may not be necessary; however, in cases with atypical manifestations or ambiguous serological results, additional molecular confirmation may be beneficial.

PCR enables direct detection of *B. henselae* DNA from clinical specimens and is especially useful when serological results are inconclusive. In countries such as Japan, where turnaround times for serological testing may be prolonged, PCR has the potential to facilitate earlier diagnosis in selected cases. In this case, the timing of PCR testing was influenced by practical limitations within the Japanese healthcare system. PCR testing for *B. henselae* is not reimbursed under public health insurance and is not routinely available in many institutions, requiring direct coordination with external laboratories. These logistical barriers increased the threshold for testing and contributed to the delayed implementation of PCR. Although not covered by public health insurance in Japan, PCR provides results within two to three days and can be performed on aspirates or tissue biopsies. In a study by Allizond et al., diagnostic sensitivity was 27% for PCR alone, 28% for IFA alone, and 44% when both were combined [6]. Positivity rates vary by specimen type, with abscess material providing the highest detection rate, while serum samples have a higher false-negative rate [10]. In this case, the timing of PCR testing was influenced by practical limitations within the healthcare system in Japan. PCR testing for *B. henselae* is not reimbursed under public health insurance and is not routinely available in many institutions, requiring direct coordination with external laboratories. These logistical barriers increased the threshold for testing and contributed to the delayed implementation of PCR.

Approximately 30% of CSD cases develop suppurative lymphadenitis; however, because abscess formation may take time, test sensitivity can vary depending on the timing of sample collection [1]. Therefore, combining serological and molecular methods is recommended to improve diagnostic accuracy, particularly in cases involving atypical anatomical sites, chronic animal exposure, or unclear onset. This case underscores the diagnostic value of PCR in confirming CSD when serological testing alone is inconclusive.

The differential diagnosis for CSD includes bacterial lymphadenitis, atypical mycobacterial infections, malignant lymphoma, and sarcoidosis [1,11]. In treatment-resistant or atypical cases, invasive diagnostic procedures such as biopsy should be considered to exclude other conditions.

In the present case, azithromycin was initiated when CSD was suspected, as it is a reasonable and widely accepted therapeutic option. Its efficacy has been demonstrated in a prospective randomized, double-blind, placebo-controlled trial by Bass et al. [12], which showed that a five-day course of oral azithromycin reduced lymph node (abscess) size in patients with CSD. Persistent fever was considered more likely to reflect suppurative or granulomatous inflammation rather than antimicrobial resistance; however, ongoing symptoms after aspiration and the development of new lymphadenopathy complicated assessment of the treatment response. In this case, multiple changes in antibiotics and repeated drainage procedures failed to produce improvement; PCR positivity in lymph node tissue and aspirated pus ultimately led to a definitive diagnosis.

From an antimicrobial stewardship perspective, early diagnostic confirmation, particularly by PCR of aspirated fluid or tissue when serological interpretation is challenging, is important to avoid prolonged empiric use of broad-spectrum antibiotics. Needle aspiration plays a dual role by providing symptomatic relief and diagnostic material, whereas incision and drainage or lymph node excision are generally discouraged because of the risk of fistula formation and should be reserved for cases with clear clinical indications.

Although PCR testing for *B. henselae* is not currently reimbursed under Japan’s public health insurance and is not routinely accessible, this resource limitation should be considered when interpreting real-world diagnostic pathways. While PCR confirmation in this case did not directly alter antimicrobial therapy, earlier diagnosis might have prevented escalation to ineffective antibiotics and reduced the need for rehospitalization or progression to invasive procedures, particularly lymph node biopsy.

While most cases of CSD resolve without intervention, atypical presentations may require advanced diagnostic methods such as PCR. Early incorporation of PCR in selected cases may streamline diagnosis, support appropriate antimicrobial use, and reduce unnecessary invasive interventions. This case highlights the importance of flexible diagnostic strategies and the timely use of molecular tools in managing CSD.

## Conclusions

We report a rare pediatric case of a CSD presenting as a treatment-resistant femoral lymph node abscess. Despite multiple courses of antibiotics, the clinical course was prolonged with recurrent episodes, making diagnosis challenging. Serological testing was confounded by elevated IgG titers from chronic cat exposure and an atypical presentation, limiting its reliability. In contrast, PCR testing complemented serology and enabled a definitive diagnosis. This case underscores the need to consider CSD in pediatric patients with atypical suppurative lymphadenitis and underscores the value of early PCR testing to guide appropriate treatment and avoid unnecessary interventions.
